# Measuring pain in non-verbal critically ill patients: which pain instrument?

**DOI:** 10.1186/s13054-014-0554-5

**Published:** 2014-10-15

**Authors:** Jean-Francois Payen, Céline Gélinas

**Affiliations:** Department of Anaesthesia and Critical Care, Michallon Hospital, bd de la Chantourne, Grenoble, F-38000 France; Joseph Fourier University, Grenoble Institute of Neurosciences, Chemin Fortuné Ferrini, Grenoble, F-38042 France; INSERM, U836, Chemin Fortuné Ferrini, Grenoble, F-38042 France; Ingram School of Nursing, McGill University, 3506 University Street, Montréal, QC H3A 2A7 Canada; Centre for Nursing Research and Lady Davis Institute, Jewish General Hospital, 3755 Cote Sainte Catherine Road, Montréal, QC H3T 1E2 Canada

## Abstract

Pain is experienced by many critically ill patients. Although the patient’s self-report represents the gold-standard measure for pain, many patients are unable to communicate in the ICU. In this commentary, we discuss the study findings comparing three objective scales for the assessment of pain in non-verbal patients and the importance of the tool selection process.

In the previous issue of *Critical Care*, Chanques and colleagues [1] evaluate the psychometric properties of three behavioral pain scales validated for use in non-communicative critically ill patients. The authors compare two scales recommended in the practice guidelines for pain management of adult ICU patients by the Society of Critical Care Medicine [2] - that is, the Behavioral Pain Scale (BPS) [3] and the Critical-Care Pain Observation Tool (CPOT) [4] - and a routine scale in use at the host institution, the Non-Verbal Pain Scale (NVPS) [5].

Assessing pain in non-communicative adult patients in the ICU must rely on the observation of behavioral indicators of pain. Selection of pain assessment tools in clinical practice must be done with rigor. Indeed, an assessment tool can be shown to be valid only for a specific purpose and a given group of respondents and context of care. All steps of scale development are important. The first step, selection of items and scale scoring, can be done by using a combination of various strategies, including an in-depth literature review, consultation of end users (for example, ICU clinicians and patients), and direct clinical observation and other sources. Content validation is a method of examining the content and relevance of the items that are useful for selecting or revising them. Once developed, reliability and validity of the scale use must be tested with the targeted patient group [6]. Reliability refers to the overall reproducibility of scale scores. The examination of inter-rater reliability is crucial to determine whether two or more trained raters reach similar scores using the same scale for the same patient and at the same time. Validity refers to the interpretation of the pain scale scores and its ability to indicate that the individual is actually in pain. As behavioral pain scales aim to detect the presence of significant pain, the examination of criterion and discriminant validation is necessary. Criterion validation allows the comparison between behavioral scores and the gold standard (that is, the patient’s self-report of pain). Discriminant validation refers to the ability of the pain scale to discriminate between conditions or procedures known to be painful or not and its ability to detect significant changes over time (responsiveness). Because validation is an ongoing process, it is imperative that its use be evaluated by independent groups of caregivers who were not involved in its development, with various ICU patient groups or with a translated version of the scale. Finally, the ease of their implementation in ICU settings and the impact of their use on pain management practices and patient outcomes must be evaluated.

Evaluation of the psychometric properties of behavioral pain scales in ICU patients unable to self-report has been recently performed [7,8]. Of the eight pain scales developed for adult ICU patients, the BPS and the CPOT were found to be the most valid and reliable. The present study [1] is the first to compare psychometric properties of these two pain scales in addition to the NVPS, at rest and during noxious (for example, turning and endotracheal suctioning) and non-noxious (for example, simple repositioning) procedures. Both the BPS and the CPOT showed the strongest psychometric properties in both intubated and non-intubated patients in comparison with the NVPS. These findings add arguments to the recommendations for the use of these two pain scales [2].

What are the next steps in relation to pain assessment in the ICU? First, there is a clear need to better evaluate the impact of pain assessment and management on patient outcomes. Few studies have shown that evaluating pain was associated with positive outcomes such as a shorter duration of mechanical ventilation and ICU length of stay and reduced adverse events [9-11]. Whether better management in the ICU may lead to reducing long-term negative consequences such as chronic pain and symptoms of post-traumatic stress disorder remains largely unknown. Second, there is a need for valid physiologic measures of pain, especially in ICU patients too sedated or paralyzed in whom behavioral responses cannot be observed. The use of pupillary reflex dilation has shown some promising findings [12-14]. Meanwhile, the best alternative measure to assess pain in non-verbal patients remains the use of behavioral scales.

Assessing pain in non-communicative ICU patients is challenging. The BPS and the CPOT have shown the strongest psychometric properties for this purpose. These scales should be incorporated into pain management protocols to target the desired levels of analgesia in order to optimize inter-professional practices and to achieve better patient outcomes.

## Abbreviations

BPS: Behavioral pain scale
CPOT: Critical-care pain observation tool
NVPS: Non-verbal pain scale
